# Pathological correlates of white matter hyperintensities in a case of progranulin mutation associated frontotemporal dementia

**DOI:** 10.1080/13554794.2018.1506039

**Published:** 2018-08-16

**Authors:** Ione O.C. Woollacott, Martina Bocchetta, Carole H. Sudre, Basil H. Ridha, Catherine Strand, Robert Courtney, Sebastien Ourselin, M. Jorge Cardoso, Jason D. Warren, Martin N. Rossor, Tamas Revesz, Nick C. Fox, Janice L. Holton, Tammaryn Lashley, Jonathan D. Rohrer

**Affiliations:** a Dementia Research Centre, Department of Neurodegenerative Disease, UCL Institute of Neurology, London, UK; b Translational Imaging Group, Centre for Medical Image Computing, University College London, London, UK; c NIHR Queen Square Dementia Biomedical Research Unit, UCL Institute of Neurology, London, UK; d Queen Square Brain Bank for Neurological Disorders, Department of Molecular Neuroscience, UCL Institute of Neurology, London, UK

**Keywords:** Frontotemporal dementia, microglia, MRI, neuroinflammation, progranulin, white matter

## Abstract

White matter hyperintensities (WMH) are often seen on MRI brain scans in frontotemporal dementia (FTD) due to progranulin (*GRN*) mutations, but their pathological correlates are unknown. We examined the histological changes underlying WMH in a patient with *GRN* mutation associated behavioral variant FTD. *In vivo* and cadaveric MRI showed progressive, asymmetric frontotemporal and parietal atrophy, and asymmetrical WMH predominantly affecting frontal mid-zones. We first performed segmentation and localization analyses of WMH present on cadaveric MRI FLAIR images, then selected five different brain regions directly matched to differing severities of WMH for histological analysis. We used immunohistochemistry to assess vascular pathology, degree of spongiosis, neuronal and axonal loss, TDP-43, demyelination and astrogliosis, and microglial burden and morphology. Brain regions with significant WMH displayed severe cortical and white matter pathology, and prominent white matter microglial activation and microglial dystrophy, but only mild axonal loss and minimal vascular pathology. Our study suggests that WMH in *GRN* mutation carriers are not secondary to vascular pathology. Whilst cortical pathology induced axonal degeneration could contribute to white matter damage, individuals with *GRN* mutations could develop selective white matter vulnerability and myelin loss due to chronic, regional microglial dysfunction arising from GRN haploinsufficiency.

## Introduction

Cerebral white matter hyperintensities (WMH) reflect an abnormal tissue fat/water ratio within myelinated brain areas, and are visible as hyperintense lesions on T2-weighted and fluid-attenuated inversion recovery (FLAIR) MRI sequences (Wardlaw, Valdés Hernández, & Muñoz-Maniega, 2015). WMH appear in healthy aging as periventricular and deep white matter lesions associated with demyelination, microgliosis and vacuolation (Murray et al., 2012), and in patients with small vessel cerebrovascular disease, associated with white matter axonal loss, demyelination, gliosis, and arteriosclerosis (Wardlaw et al., 2015). WMH also occur in neuroinflammatory diseases such as multiple sclerosis (Sarbu et al., 2016), and in neurodegenerative diseases (Filley & Fields, 2016). Research into the pathological correlates of WMH in vascular dementia and sporadic and familial Alzheimer’s disease (AD) is well-established (McAleese et al., 2015, 2017; Ryan et al., 2015; Snyder et al., 2015). However, WMH are less commonly seen in frontotemporal dementia (FTD). Several studies have now reported prominent WMH in patients with familial FTD due to progranulin (*GRN*) mutations (Caroppo et al., 2014; Ameur et al., 2016; Kelley et al., 2009; Le Ber et al., 2008; Paternicò et al., 2016; Pietroboni et al., 2011; Sudre et al., 2017) but the pathological changes underlying these have not previously been studied in detail.

In order to establish accurately the pathological correlates of WMH, precise correlation of neuroimaging abnormalities with histological changes in corresponding brain tissue is needed. This was previously difficult to establish through use of *in vivo* MRI in patients who later came to post-mortem, as WMH evident on the last available *in vivo* scan typically did not match the location and severity of brain tissue abnormalities seen after death. However, use of cadaveric brain MRI enables scanning of the intact post-mortem brain *in situ* within 24 h of death (Tofts et al., 2008). It provides detailed, undistorted images of the brain, avoiding the changes that can occur with post-mortem scanning following brain removal and fixation, and allows precise spatial and quantitative correlations of neuroimaging abnormalities with histological findings. We therefore used this technique to examine the pathological correlates of WMH in a patient with *GRN* mutation associated FTD. We first characterized the location and burden of WMH present on cadaveric MRI then selected several different regions of brain tissue for detailed histological examination, which corresponded closely with presence of differing severities of WMH. In addition to assessing pathology identified in previous studies of WMH in neurodegnerative disease, we carried out a comprehensive assessment of vascular pathology using recent consensus guidelines (Skrobot et al., 2016), and, given the role of GRN in microglial function, examined patterns of several different microglial markers.

## Case report

A 60 year-old left-handed female developed progressive behavioral change, speech disturbance and episodic memory impairment. Initially she developed prominent apathy, reduced social interaction, sweet tooth, disinhibition, and new obsessions, followed by reduced speech output and use of stereotypic phrases. Two years after onset she had difficulty remembering recent events and conversations, and got lost in familiar places, then developed dyscalculia. She had occasional visual hallucinations of family members. Her condition rapidly worsened four years after onset and she was admitted to a nursing home with impaired spatial awareness, prosopagnosia, wandering and incontinence. She later developed an asymmetrical extrapyramidal syndrome with dystonic limb movements, worse on the right. She died aged 68 with a final clinical diagnosis of behavioral variant FTD. The patient donated her brain for post-mortem analysis to the Queen Square Brain Bank for Neurological Disorders (QSBB) at University College London Institute of Neurology.

The patient’s medical history included progressive right-sided facial hemi-atrophy, diagnosed 15 years prior to onset of cognitive symptoms, with no cause identified. Two years after onset of cognitive symptoms she developed a persistent cough and peripheral eosinophilia, pericardial thickening and an anterior pericardial effusion. The cause of these symptoms was not identified despite thorough investigation, but was felt to be autoimmune in origin. There were no vascular risk factors, no prior smoking history, and consistently normal blood pressure. Her mother had developed acute psychosis with delusions and odd behavior aged 65, followed by progressive cognitive decline and admission to a mental health unit for 20 years, with an eventual diagnosis of dementia. There was no other family history of dementia or neurological or vascular disease.

Investigations included a volumetric 1.5 T MRI brain performed 2.6 years before the patient died. This showed bilateral but asymmetric frontal, temporal, and parietal atrophy, worse on the left, and significant bilateral periventricular WMH, worst in the frontal lobes and on the left side. Cerebrospinal fluid analyses were normal or negative. A wide variety of blood tests were normal, except for peripheral eosinophilia of 1.11 (range 0.0–0.4 x 10^9^/L) and positive rheumatoid positive particle agglutination test (1/80 titer). DNA analysis identified a heterozygous *GRN* Q130fs mutation but no other FTD-associated mutations.

## Methods

### Imaging analysis

The *in vivo* 1.5 T MRI was performed on a GE Signa scanner including volumetric T1, and T2, but not FLAIR, sequences. The patient had also consented to participation in a cadaveric MRI study, approved by the National Hospital for Neurology and Neurosurgery and Institute of Neurology Joint Research Ethics Committee. A 1.5 T GE Signa MRI scanner was used within 24 h of her death to perform cadaveric *in situ* brain MRI, including volumetric T1 and FLAIR sequences, as described previously (Tofts et al., 2008).

The *in vivo* and cadaveric MRI volumetric T1 images were compared using the Statistical Parametric Mapping (SPM)12 Serial Longitudinal Registration tool (www.fil.ion.ucl.ac.uk/spm) to estimate the percentage of volumetric contraction for each voxel between the two scans and assess degree of brain atrophy over time. Although WMH were present on both the *in vivo* and cadaveric MRI, only the cadaveric MRI could be used for further imaging analysis, due to lack of *in vivo* FLAIR and poor quality *in vivo* T2-weighted imaging. This precluded quantitative comparison of interval change in WMH between scans. Regional quantification of WMH in the cadaveric MRI FLAIR sequence was performed using segmentation and localization analyses as previously described (Sudre et al., 2017, 2015; Sudre, Cardoso, & Ourselin, 2017) (see Supplementary Material). This allowed quantification of WMH burden in each lobe and within different cortical and subcortical “layers”, guiding selection of regions for histological analysis.

### Immunohistochemistry and histological analysis

The brain donation protocol at QSBB was approved by a London Research Ethics Committee, and the tissue stored for research purposes under a license from the Human Tissue Authority. The right hemisphere was fixed and left hemisphere frozen as per standard protocol, and the right hemisphere cut into 5mm thick coronal slices. Paraffin-embedded tissue sections from a large number of brain regions underwent routine post-mortem analysis, including examination for tau, transactive response DNA binding protein 43 (TDP-43), alpha-synuclein and amyloid beta (Aβ) deposition, neuronal, myelin and axonal loss and astrogliosis (Lashley et al., 2008; Ryan et al., 2015). The circle of Willis was examined for presence of atheroma, and other relevant cortical, subcortical and hippocampal regions (including leptomeningeal vessels) were examined for vascular pathology.

**Figure 1.**
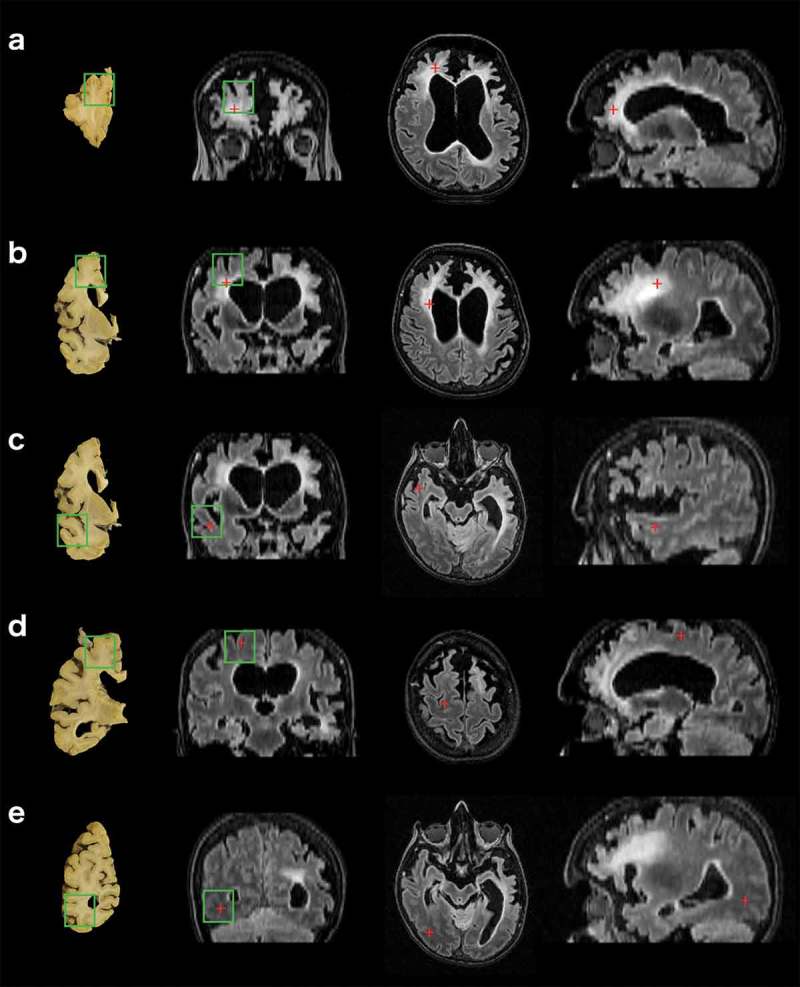
Comparison of brain tissue slices with FLAIR sequences of cadaveric MRI scan to enable accurate tissue block selection. (a) right frontal pole, (b) right anterior frontal lobe, (c) right temporal lobe, (d) right posterior frontal lobe and (e) right occipital lobe. Images in the far left column show where tissue blocks were obtained from post-mortem brain tissue slices. Right three columns show, from left to right: cadaveric brain MRI images in the coronal, axial, and sagittal planes. Green boxes indicate borders of the tissue block cut from this region, superimposed onto the corresponding tissue slice and MRI image. Red crosses represent the same anatomical point viewed across all three planes.

Five brain regions were selected for detailed histological analysis to examine the pathological correlates of differing severities of WMH. First, we identified brain tissue slices from the fixed right hemisphere that anatomically matched relevant cadaveric MRI FLAIR image slices on coronal, sagittal and axial views (Figure 1). Then we selected five brain regions corresponding to the following degrees of WMH severity, based on both visual inspection and imaging analyses of cadaveric MRI FLAIR images: right frontal pole (severe; Figure 1(a)), anterior frontal lobe (moderate to severe; Figure 1(b)), temporal lobe (mild to moderate; Figure 1(c)), posterior frontal lobe (very mild; Figure 1(d)), and occipital lobe (absent/none; Figure 1(e)). Paraffin-embedded sections were cut from tissue blocks obtained from each of these regions (Figure 1). Sections were stained with hematoxylin and eosin (H&E), Luxol fast blue (LFB) and Perl stains to assess the degree of neuronal loss and spongiosis, myelin loss, and hemosiderin deposition. Immunohistochemistry was performed to detect the presence of TDP-43, Aβ, tau, alpha synuclein, phosphorylated neurofilaments (axons) and activated astrocytes (see Supplementary Material).

We performed further immunohistochemistry (see Supplementary Material) on all five regions to investigate whether regions with severe WMH were associated with different degrees of microglial burden, activation or morphology, compared with regions where WMH were milder or absent. Three antibodies were used to detect microglia with differing phenotypes, in both ramified and activated states: cluster of differentiation CD68 (CD68), which detects a lysosomal and endolyosomal glycoprotein upregulated in activated, phagocytic microglia; CR3/43, which detects human leucocyte antigen-DR (HLA-DR), a molecule expressed by microglia with antigen presenting cell function and upregulated in inflammation; and ionized calcium-binding adapter molecule 1 (Iba1), which is expressed by resting microglia but upregulated on activated microglia, may reflect microglial motility, and is useful for assessing microglial morphology (Minett et al., 2016; Streit, Braak, Xue, & Bechmann, 2009; Tischer et al., 2016).

Grey and white matter from the five brain regions were assessed for histological changes through microscopic examination and consensus opinion of two neuropathologists blinded to the severity of WMH. Sections were qualitatively assessed for degree of neuronal loss and spongiosis and semi-quantitatively assessed for degree of axonal loss, myelin loss, TDP-43, astrogliosis, and presence of microglia (CD68, CR3/43 and Iba1 positive cells). Semi-quantitative assessment used a multi-tier scoring system modified from a previous study (Ryan et al., 2015), where: ‘0ʹ represents no axonal loss, no white matter pallor, no TDP-43 present, no astrogliosis or no visible microglia; “+” or ‘1ʹ represents a mild degree of axonal loss, pallor of the white matter, TDP-43 burden, astrogliosis or presence of few microglia; “++” or ‘2ʹ represents a moderate degree of these changes or presence of moderate numbers of microglia; and “+++” or ‘3ʹ represents a severe degree of these changes or presence of many microglia. In addition, qualitative descriptions of microglial morphology were recorded, for example whether microglia appeared amoeboid (round), suggestive of microglial activation, or ramified (with extended processes), or any other atypical morphological appearances. Axonal and myelin loss were scored in white matter of each region, neuronal loss and spongiosis in grey matter, and both grey and white matter within each region were assessed for other histological changes, but only scored separately for microglial markers, or if significant differences in other histological changes were present.

Vascular pathology was semi-quantitatively assessed in grey and white matter of all five regions using a comprehensive scoring system based on the consensus recommendations of the Vascular Cognitive Impairment Neuropathology Guidelines (VCING) for staging of cerebrovascular pathology in cognitive impairment (Skrobot et al., 2016) (see Supplementary Material). Leptomeningeal vessels were also examined for presence of atheroma in all five brain regions. Presence of atheroma in the circle of Willis and vascular changes in other cortical and subcortical areas had been examined during routine post-mortem. Although the VCING include scores for myelin loss, all regions were also scored for white matter pallor using our modified multi-tier scoring system (Ryan et al., 2015) to allow direct comparison between this score of myelin loss and scores for non-vascular histological changes.

## Results

### Imaging findings

**Figure 2.**
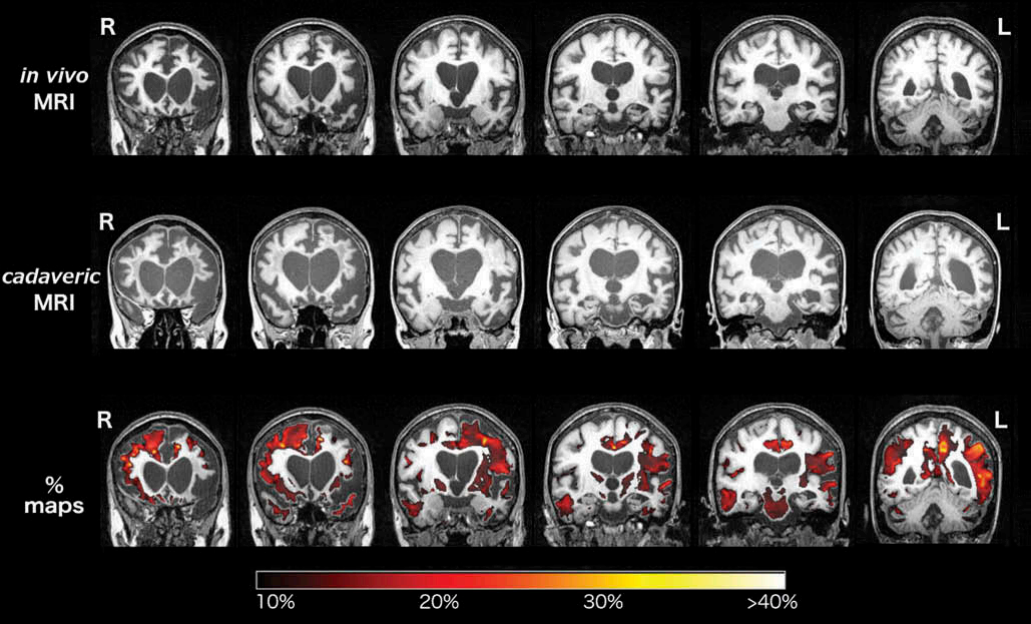
Coronal sections of the *in vivo* (top row) and cadaveric (second row) T1-weighted MRI brain scans in the anterio-posterior direction. The third row shows the longitudinal Statistical Parametric Mapping (SPM) overlay representing 10% or greater volumetric contraction of the brain on the cadaveric scan, compared with the *in vivo* scan, to show interval atrophy, with corresponding color bar scale.

**Figure 3.**
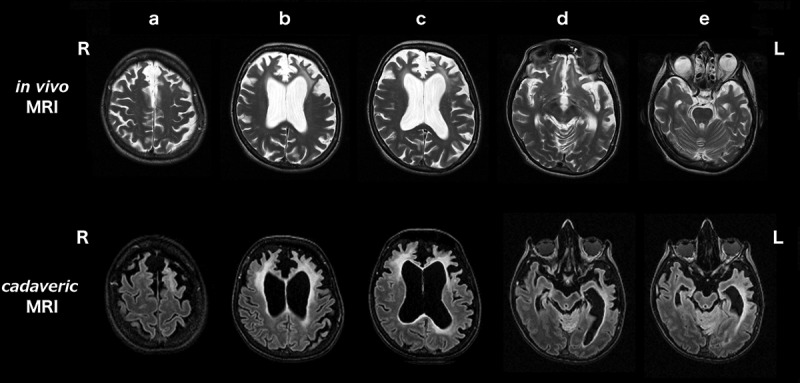
Axial sections of the *in vivo* T2-weighted (top row) and cadaveric FLAIR (second row) MRI brain scans. Scans show the change in severity and distribution of WMH in the 2.6 year interval between scans, in the rostro-caudal direction from (a) to (e). (a) posterior frontal lobes, (b) anterior frontal lobes, (c) frontal poles, (d) occipital lobes and (e) temporal lobes. R right and L left side of patient.

**Figure 4.**
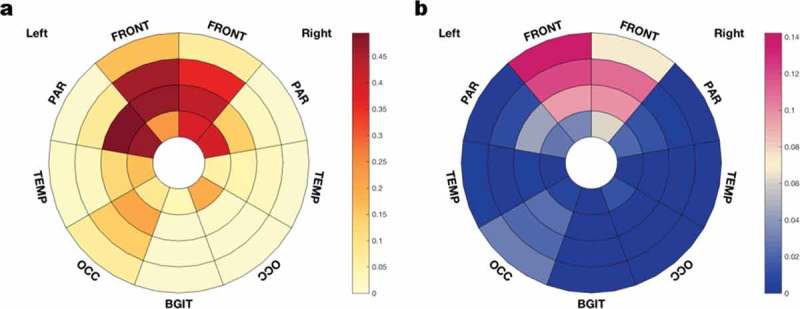
Bull’s-eye schematic of observed WMH lesion frequency (a), and lesion distribution (b), within different brain regions and layers in the cadaveric MRI brain scan. Each region of the brain is apportioned into four layers, with the ventricle represented at the center of the diagram and the cortical sheet at the external edge. Lesion frequency is the proportion of the volume of a brain region taken up by WMH. Lesion distribution is the proportion of the total brain WMH lesion load present in that region. Degree of lesion frequency and distribution are represented using a color scale bar. FRONT frontal lobes, PAR parietal lobes, TEMP temporal lobes, OCC occipital lobes, BGIT basal ganglia and thalami.

The *in vivo* MRI brain scan showed asymmetric (left worse than right) frontal, temporal and parietal atrophy (Figure 2). Between the *in vivo* and cadaveric MRI there had been volume loss, particularly affecting left posterior frontal and parietal, and right frontal, areas (Figure 2 bottom panel). WMH were present asymmetrically on both *in vivo* T2 and cadaveric FLAIR images (Figures 1 and 3), predominantly affecting both frontal lobes, as confirmed by the localization analysis of cadaveric FLAIR images (Figure 4). WMH were most severe in the left frontal pole and anterior frontal lobe (Figures 1, 3, and 4), matching areas of most significant cortical atrophy (Figures 2 and 3), although were also severe in the right frontal pole and anterior frontal lobe. WMH were present predominantly in the intermediate layers (layers two to three) of the frontal lobes, rather than immediately periventricularly or juxtacortically (Figure 4). WMH were also seen in the parietal lobes (more on the left than the right), and to a much lesser extent in the posterior frontal and temporal lobes, with minimal or no WMH in the occipital lobes (Figures 1, 3 and 4).

### Histological changes and correlation with imaging

Routine post-mortem analysis revealed severe neuronal loss and spongiosis, particularly in the anterior frontal, temporal and insular cortices and caudate, and severe myelin loss and gliosis in the frontal and temporal lobes. There was also severe myelin loss in the external capsule, extreme capsule and anterior internal capsule. There was comparatively milder axonal loss in the frontal and temporal lobes. The cerebellum and occipital lobes had minimal pathology. Inclusions of TDP-43 were present in neuronal cytoplasm and occasional neuronal nuclei, together with TDP-43 positive neuropil threads, particularly in frontal lobe grey and white matter, consistent with frontotemporal lobar degeneration with TDP (FTLD-TDP) type A pathology (Mackenzie et al., 2011). There was no Aβ, tau or alpha-synuclein pathology. There was no atheroma in the vast majority of leptomeningeal vessels in cortical and subcortical regions (very rare vessels had atheroma with < 50% vessel occlusion), and no other vascular pathology. There was only mild (grade 1) atheroma in the circle of Willis.

**Figure 5.**
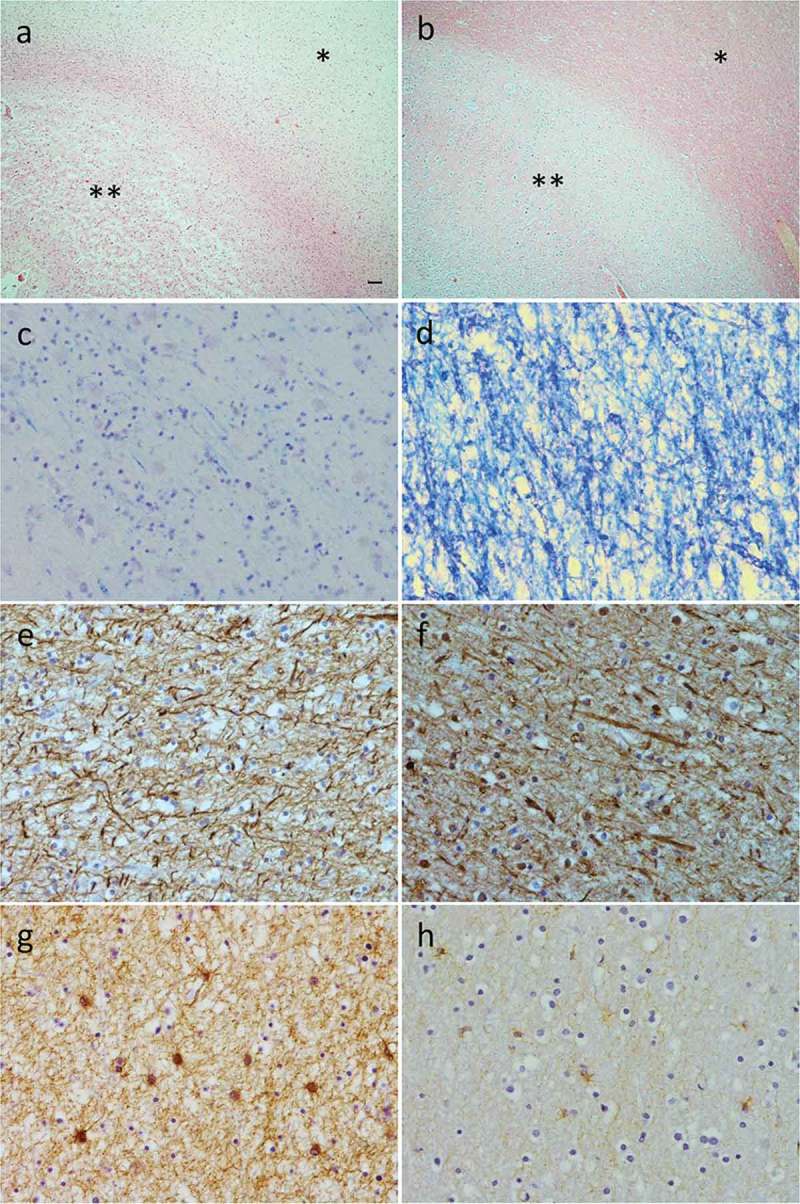
Histological changes in a brain region with severe WMH (right frontal pole; a, c, e, and g) and in a region with no WMH (right occipital lobe; b, d, f and h). Hematoxylin and eosin staining demonstrates severe grey matter spongiosis (**) and pallor of the white matter (*) in the frontal pole (a). In the occipital lobe (b) there is intact cytoarchitecture of the grey matter (**) and white matter (*). Luxol fast blue staining demonstrates extensive myelin pallor in the white matter of the frontal pole (c) but intact myelin in the occipital lobe (d). SMI31 immunohistochemistry demonstrates relative preservation of axons in the white matter of both frontal pole (e) and occipital lobe (f). Severe gliosis was present in frontal pole white matter (g) but only mild in occipital lobe white matter (h). Scale bar in (a) represents 100µm for (a) and (b), and 10µm for (c) to (h).

**Figure 6.**
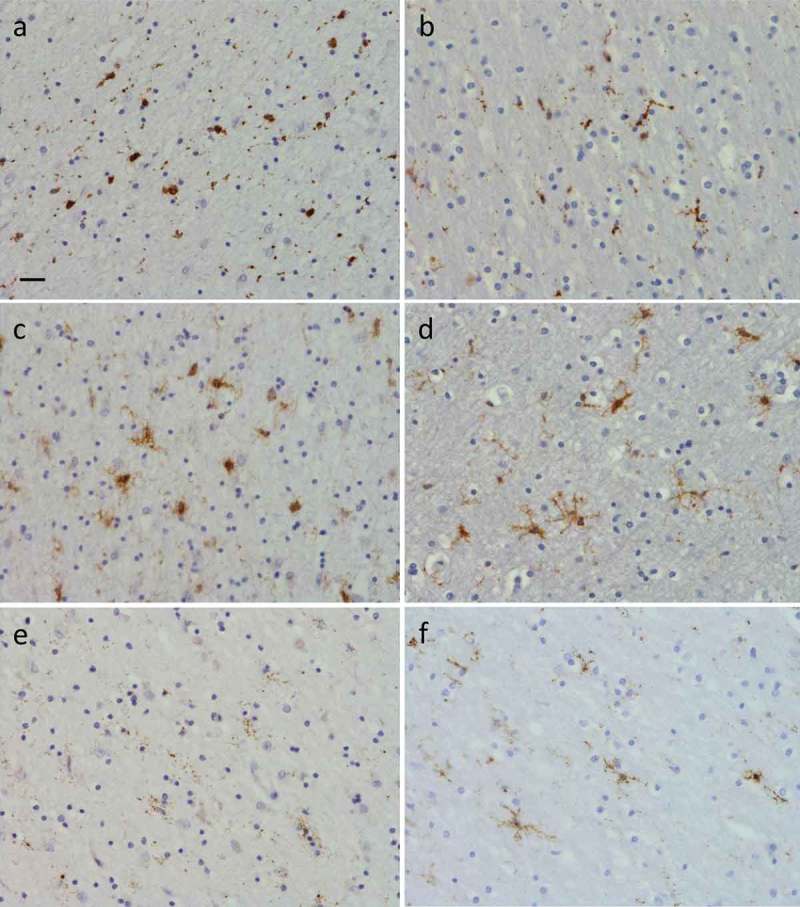
Presence of microglia in right frontal pole white matter (a), (c) and (e), where there were severe WMH, and right occipital white matter (b), (d) and (f), where WMH were absent. Images show results of immunohistochemistry for CD68 (a) and (b), CR3/43 (c) and (d) and Iba1 (e) and (f) positive microglia. There were more amoeboid microglia in the frontal versus occipital white matter detected using CD68 (a) versus (b), and CR3/43 (c) versus (d). Iba1 positive microglia were ramified in occipital white matter (f) but few in number and severely dystrophic in frontal white matter (e). Scale bar in (a) represents 20µm in all panels.

Results of detailed regional histological analyses are summarized in Table 1, Table 2, Figures 5 and 6. Brain regions with the most severe WMH (frontal pole and anterior frontal lobe) displayed severe white matter pathology, including myelin pallor and astrogliosis (Table 1; Figure 5(c) and (g)) and severe TDP-43 burden (Table 1) but no or minimal vascular pathology (Table 1) and relative preservation of axons (Figure 5(e)). Regions with very mild or absent WMH, such as the posterior frontal lobe or occipital lobe, generally had very mild or no histological changes in both grey and white matter (Figure 5 (b, d, f, h)). Except for myelin loss on LFB staining, there were minimal features of vascular pathology in all regions analyzed (Table 1). There was very mild arteriolosclerosis present in occasional white matter vessels in all five brain regions of interest but no atheroma in leptomeningeal vessels. Brain regions with the most severe WMH on MRI also displayed the most severe cortical (grey matter) pathology, including neuronal loss, spongiosis, astrogliosis, and TDP-43 burden (Table 1). The severity of cortical pathology corresponded with the degree of white matter pathology in all regions analyzed, with the exception of the frontal pole (Figure 5(e)), anterior frontal lobe and temporal lobe, which displayed relatively mild axonal loss despite more severe degrees of other grey and white matter pathology.

**Table 1.** Histological changes assessed in five brain regions with differing severities of WMH. Scores: + mild, ++ moderate, +++ severe; (0–3) or (0–4) as stated in table, or in Supplementary Material Table 1. CAA, cerebral amyloid angiopathy; GM, grey matter; WM, white matter.

**Brain region**	**Right frontal pole**	**Right anterior frontal lobe**	**Right temporal lobe**	**Right posterior frontal lobe**	**Right occipital lobe**
**WMH severity on MRI**	Severe	Moderate to severe	Mild to moderate	Very mild	None
**Cortical spongiosis** (None/very mild/mild/moderate/severe/full thickness)	Full thickness; U fibers preserved	Full thickness; U fibers preserved	Severe	Very mild	None
**Cortical neuronal loss** (No loss/mild/moderate/severe)	Severe	Severe	Severe	Mild	No loss
**White matter myelin pallor** (0 = none; 1 = mild; 2 = moderate; 3 = severe)	3	3	3	0	0
**White matter axonal loss** (0 = none; 1 = mild; 2 = moderate; 3 = severe)	1	1	1	0	0
**Astrogliosis** (0 = none; 1 = mild; 2 = moderate; 3 = severe)	GM: 3 WM:3	GM:3 WM: 3	GM: 1 WM: 3	GM: 1 WM: 3	GM: 1 WM:1
**TDP-43 (Type A)** **(0/+/++/+++)**	++ Moderate cytoplasmic and nuclear inclusions plus threads	++/+++ Moderate cytoplasmic and nuclear inclusions plus threads	+ Sparse nuclear inclusions, a few threads	+ Sparse nuclear inclusions, a few threads	+ Only a few threads
**Myelin loss** (0–3)	3	3	3	0	0
**Arteriolosclerosis** (0–3)	1	1	1	1	1
**Fibrinoid necrosis** (0/1)	0	0	0	0	0
**Microaneurysms** (0/1)	0	0	0	0	0
**Perivascular space dilation** (0–3)	1	2	2	1	0
**Perivascular hemosiderin leakage** (0–3)	0	1	0	0	0
**Microinfarcts** (0/1)	0	0	0	0	0
**Lacunar infarcts** (0–3)	0	0	0	0	0
**Large infarcts** (0/1)	0	0	0	0	0
**Microhemorrhage** (0/1)	0	0	0	0	0
**Larger hemorrhage** (0/1)	0	0	0	0	0
**Leptomeningeal CAA** (0–4)	0	0	0	0	0
**Cortical CAA** (0–4)	0	0	0	0	0
**Capillary CAA** (0/1)	0	0	0	0	0

**Table 2.** Microglial burden and morphology in five brain regions with different severities of WMH on MRI. “Amoeboid”: majority of microglia were round. “Ramified”: majority of microglia had branched processes. “Amoeboid and ramified”: a mixture of both morphologies. “Dystrophic”: punctate staining of microglial cell processes producing a fragmented, beaded appearance. **+** few microglia, **++** moderate numbers of microglia, **+++ **many microglia.

	**Brain region**	**Right frontal pole**	**Right anterior frontal**	**Right temporal**	**Right posterior frontal**	**Right occipital**
Microglial marker	WMH severity	Severe	Moderate to severe	Mild to moderate	Very mild	Absent
CD68	Grey matter	+ Amoeboid	++ Amoeboid	+ Amoeboid & Ramified	+ Ramified	+ Ramified
	White matter	++ Amoeboid	+++ Amoeboid	+++ Amoeboid & Ramified	+++ Amoeboid & Ramified	++ Ramified
CR3/43	Grey matter	+ Amoeboid	+ Amoeboid	++ Amoeboid	++ Amoeboid & Ramified	+ Ramified
	White matter	++ Amoeboid	+++ Amoeboid	+++ Amoeboid & Ramified	+++ Ramified	++ Ramified
Iba1	Grey matter	+++ Amoeboid	+++ Amoeboid	++ Amoeboid & Ramified	++ Amoeboid	+ Ramified
	White matter	+ Dystrophic	+ Dystrophic	+ Dystrophic	+ Ramified, few dystrophic	+ Ramified

### Patterns of microglia

Microglial burden, activation state and morphology differed between regions with severe WMH (frontal lobe), and those without WMH (occipital lobe) (Table 2; Figure 6). There were generally more CD68, CR3/43 and Iba1 positive microglia present in frontal and temporal grey matter than occipital grey matter (Table 2). There were also more CD68 (Figure 6(a)) and CR3/43 (Figure 6(c)) positive microglia present in frontal than occipital white matter (Figure 6 (b) and (d)), but similarly few Iba1 positive microglia in white matter of both regions (Figure 6(e) and (f)). Although there were generally more CD68 and CR3/43 positive microglia present in white matter than in grey matter of all regions, there were much fewer Iba1 positive microglia in frontal and temporal white matter than grey matter (Table 2). In the occipital lobe, the burden of Iba1 positive microglia was similar in grey and white matter. In terms of activation state and morphology, CD68 and CR3/43 positive microglia were amoeboid in appearance in frontal lobe grey and white matter (Table 2; Figure 6(a) and (c)), but ramified in occipital grey and white matter (Table 2; Figure 6(b) and (d)). There was a mixture of amoeboid and ramified CD68 and CR3/43 positive microglia in regions with intermediate WMH severity (posterior frontal or temporal lobe; Table 2). Iba1 positive microglia were also amoeboid in appearance in frontal grey matter (Table 2). However, in frontal pole and anterior frontal white matter, where there were significant WMH, Iba1 positive microglia displayed punctate staining throughout microglial cell processes, producing a fragmented appearance, typical of severely dystrophic microglia (Figure 6(e)). This was in contrast to the ramified appearance of Iba1 positive microglia in occipital lobe white matter, where WMH were absent (Figure 6(f)). Dystrophic microglia were not observed in frontal grey matter (despite a similar burden of TDP-43 and other pathology) or in grey matter of any other region analyzed (Table 2). Microglia were less dystrophic in white matter within the temporal lobe (moderate WMH) and posterior frontal lobe (mild WMH).

## Discussion

White matter changes such as gliosis and demyelination have long been observed in histological studies of FTD (Englund & Brun, 1987) and several groups have observed significant WMH on MRI brain scans of *GRN* mutation carriers with FTD who lack vascular risk factors (Caroppo et al., 2014; Ameur et al., 2016; Kelley et al., 2009; Le Ber et al., 2008; Paternicò et al., 2016; Pietroboni et al., 2011; Sudre et al., 2017). However, studies that have characterized in detail the pathological correlates of WMH in *GRN* mutation associated FTD have until now been lacking. Our study used a segmentation and localization analysis of WMH present on cadaveric MRI FLAIR images of a patient with *GRN* mutation associated FTD, and careful correlation with post-mortem brain tissue, to enable analysis of the pathological correlates of WMH in both grey and white matter of several different affected and unaffected brain regions. Our histological assessment encompassed a wide range of morphological vascular changes using recent consensus guidelines (Skrobot et al., 2016), and other relevant histological changes examined in previous studies of WMH in other dementias. In addition, we analyzed the burden, activation state and morphology of three different microglial phenotypes across multiple brain regions, which have not been described previously in FTD. Through this approach we demonstrate that WMH in *GRN* mutation associated FTD occur predominantly in frontal lobe mid-zones and are associated with significant cortical pathology and white matter demyelination and gliosis, but only mild axonal loss and minimal vascular pathology. Microglia expressing Iba1 are severely dystrophic in regions with significant WMH, such as frontal white matter, suggestive of regional microglial dysfunction associated with white matter disease.

Our patient had prominent, asymmetrical WMH on both *in vivo* and cadaveric brain MRI. Imaging analysis revealed that WMH were most prominent in the middle layers of the frontal lobes, as well as being present in periventricular regions. WMH were present to a lesser degree in the parietal and temporal lobes, but were minimal or absent in the occipital lobes. This pattern of predominantly frontal WMH, extending out into the “mid-zones” of white matter areas, seems to be a particular feature of *GRN* mutation carriers. In a larger study of symptomatic and presymptomatic individuals with familial FTD, symptomatic *GRN* mutation carriers had increased global load of WMH compared with *MAPT* and *C9orf72* mutation carriers (Sudre et al., 2017). WMH were most prominent in the three layers closest to the lateral ventricles within the frontal and parietal lobes, and were more homogeneous in appearance in the *GRN* mutation group, suggesting a different aetiology from WMH in other mutation carriers (Sudre et al., 2017). Similar patterns of WMH were observed in smaller imaging studies of WMH in *GRN* mutation carriers (Caroppo et al., 2014; Ameur et al., 2016).

Through quantitative assessment of regional WMH burden and atrophy on MRI we determined that the severity of WMH correlated with the location and extent of cortical atrophy. This is consistent with correlations described in other imaging studies (Caroppo et al., 2014; Ameur et al., 2016; Kelley et al., 2009; Sudre et al., 2017). Using precise anatomical correlation of cadaveric MRI brain images with tissue slices, we were also able to demonstrate that regions with the most severe WMH displayed the most severe cortical pathology on histological analysis. The severity of white matter pathology also corresponded with the degree of cortical pathology, and WMH severity, in the majority of brain regions analyzed. This could suggest that WMH in *GRN* mutation associated FTD are due to axonal degeneration and demyelination secondary to cortical neuronal loss, induced by cortical TDP-43 aggregates. In AD, cortical hyperphosphorylated tau burden is predictive of the severity of WMH on MRI, suggesting that cortical tau pathology contributes to AD-related WMH (McAleese et al., 2015). The degree of cortical pathology, demyelination, axonal loss and Wallerian degeneration also correlates with the extent of white matter lesions in AD, suggesting a link between grey matter pathology and white matter damage (McAleese et al., 2015, 2017). In our study we observed significant cortical TDP-43 burden and neuronal loss in regions with severe WMH on MRI (such as the frontal lobe), which correlated with the severity of demyelination and white matter gliosis in the same region. However, we did not observe significant axonal loss in these regions. This suggests that myelin loss alone, rather than axonal degeneration due to cortical pathology, may have led to the appearance of WMH on MRI in our case.

An alternative hypothesis is that the prominent WMH in *GRN* mutation carriers are secondary to small vessel disease. However, through comprehensive assessment of multiple brain regions we demonstrated that there was minimal vascular pathology in our patient, despite presence of significant other grey and white matter pathology in regions with severe WMH. Our results suggest that small vessel disease does not underlie WMH in symptomatic *GRN* mutation carriers. Although mild arteriolosclerosis was present, and there was mild atheroma in rare leptomeningeal vessels in subcortical regions and the circle of Willis, mild pathological changes such as these are commonly seen incidentally in healthy aging and in dementia. In a study of the pathological correlates of WMH in a mixed cohort of cases (aged controls, AD, and individuals with significant vascular risk factors) mild arteriosclerosis was present in 56/57 cases and not significantly different between those with and without WMH (Shim et al., 2015). A lack of correlation between markers of ischaemia and small vessel sclerosis and parietal white matter lesions was observed in two studies of AD (McAleese et al., 2015, 2017). These studies, and our findings, underline the importance of not simply attributing cortical WMH on MRI as due to cerebrovascular disease when assessing patients with cognitive impairment.

Many patients with sporadic FTD and significant grey and white matter spongiosis at post-mortem do not display WMH on MRI. In addition, although white matter disease is generally more extensive in individuals with FTLD-tau than FTLD-TDP (Irwin et al., 2018; McMillan et al., 2013), MRI WMH are much less common in *MAPT* mutation carriers, who have FTLD-tau, than *GRN* mutation carriers, who have FTLD-TDP (Sudre et al., 2017). Mechanisms independent of FTLD may therefore be responsible for white matter damage in individuals with *GRN* mutations, including mutation-specific mechanisms affecting white matter vulnerability. Increasing evidence implicates GRN in regulation of neuroinflammation (Chitramuthu, Bennett, & Bateman, 2017), particularly in microglial activation and function (Lui et al., 2016). A study of two *GRN* mutation carriers observed significant microgliosis broadly corresponding to areas of WMH on MRI (Kelley et al., 2009). White matter lesions in *GRN* mutation carriers could therefore be due to chronic neuroinflammation, and we hypothesized that WMH in our *GRN* mutation carrier would be associated with regional differences in microglia. Our analysis used three different microglial phenotypic markers (CD68, CR3/43 and Iba1) to explore, in detail, differences in the burden, activation state and morphology of between brain regions with and without WMH.

We observed many amoeboid CD68 positive microglia in both grey and white matter of regions with severe WMH (frontal lobe) or moderate WMH (temporal lobe), but particularly in white matter. This is consistent with the large number of activated CD68 positive microglia in *GRN* mouse models (Lui et al., 2016; Martens et al., 2012; Yin et al., 2009, 2010) and patients with *GRN* mutations, especially in frontal white matter (Lant et al., 2014; Taipa et al., 2017), which could cause white matter damage through excessive phagocytosis. We also identified many amoeboid CR3/43 positive microglia in areas of severe WMH, suggesting that microglia with antigen-presenting cell function were particularly active in these regions, which has not been described previously in FTD. In the occipital lobe, where WMH were absent, CD68 and CR3/43 positive microglia were less numerous and more ramified, indicating a less active phenotype, consistent with minimal other pathology.

In contrast, there were fewer Iba1 positive microglia in frontal and temporal white matter than in grey matter. We also observed severely dystrophic Iba1 positive microglia (Streit et al., 2009) in frontal and temporal white matter, but not in occipital lobe white matter, correlating with the presence and location of WMH. In frontal and temporal grey matter, which displayed a similar burden of other pathology compared with white matter, Iba1 positive microglia were typically amoeboid, rather than dystrophic, in appearance. This dissociation between degree of microglial dystrophy and other pathology suggests that there may be region-specific microglial dystrophy in *GRN* mutation carriers, perhaps due to premature microglial dysfunction or senescence (Streit, Xue, Tischer, & Bechmann, 2014) secondary to GRN haploinsufficiency. As Iba1 expression may reflect microglial motility (Minett et al., 2016), microglia may be less motile in frontal white matter of *GRN* mutation carriers. A reduction in Iba1 positive microglia was observed in patients with AD using similar microglial markers (Minett et al., 2016), and Iba1 positive dystrophic microglia are present in AD cases (Streit et al., 2009; Tischer et al., 2016), preceding the spread of tau pathology (Streit et al., 2009). Impaired microglial motility or function within regions such as frontal and temporal white matter could reduce neuronal support, leading to white matter vulnerability in *GRN* mutation carriers that develop WMH. Activation of microglia with phagocytic and antigen presenting cell properties in response to burgeoning protein aggregation could exacerbate demyelination and white matter damage. This theory requires further exploration using quantitative measures of microglial function and motility, as well as burden and morphology.

## Conclusions

Presence of significant MRI WMH in a patient with symptoms of FTD, a positive family history, and no obvious vascular risk factors, particularly within frontal white matter, could signal an underlying *GRN* mutation and should prompt consideration of genetic testing (Paternicò et al., 2016). As WMH in our *GRN* mutation carrier were not associated with vascular pathology, they could instead be due to axonal degeneration secondary to cortical pathology, or region-specific white matter vulnerability, contributed to by *GRN* haploinsufficiency and microglial dysfunction. As our study included only one case, exploration of the pathological correlates of WMH across a larger cohort of patients with FTD is needed. Future work should focus on establishing patterns of grey and white matter pathology and microglial phenotypes across relevant brain regions in a larger number of *GRN* mutation cases, and in individuals with a range of different subtypes of FTLD, both with and without WMH on MRI.

## References

[CIT0001] Ameur F., Colliot O., Caroppo P., Stroer S., Dormont D., Brice A., … Bertrand A. (2016). White matter lesions in FTLD: Distinct phenotypes characterize GRN and C9ORF72 mutations. *Neurology: Genetics*, 2, e47–e47. 27066584 10.1212/NXG.0000000000000047 PMC4817905

[CIT0002] Caroppo P., Le Ber I., Camuzat A., Clot F., Naccache L., Lamari F., … Brice A. (2014). Extensive white matter involvement in patients with frontotemporal lobar degeneration. *JAMA Neurology*, 71, 1562. 25317628 10.1001/jamaneurol.2014.1316

[CIT0003] Chitramuthu B. P., Bennett H. P. J., & Bateman A. (2017). Progranulin: A new avenue towards the understanding and treatment of neurodegenerative disease. *Brain*, 140, 3081–3104. 29053785 10.1093/brain/awx198

[CIT0004] Englund E., & Brun A. (1987). Frontal lobe degeneration of non-Alzheimer type. IV. White matter changes. *Archives of Gerontology and Geriatrics*, 6, 235–243. 3689056 10.1016/0167-4943(87)90024-0

[CIT0005] Filley C. M., & Fields R. D. (2016). White matter and cognition: Making the connection. *Journal of Neurophysiology*, 116, 2093–2104. 27512019 10.1152/jn.00221.2016 PMC5102321

[CIT0006] Irwin D. J., McMillan C. T., Xie S. X., Rascovsky K., Van Deerlin V. M., Coslett H. B., … Grossman M. (2018). Asymmetry of post-mortem neuropathology in behavioural-variant frontotemporal dementia. *Brain*, 141, 288–301. 29228211 10.1093/brain/awx319 PMC5837322

[CIT0007] Kelley B. J., Haidar W., Boeve B. F., Baker M., Graff-Radford N. R., Krefft T., … Petersen R. C. (2009). Prominent phenotypic variability associated with mutations in progranulin. *Neurobiology of Aging*, 30, 739–751. 17949857 10.1016/j.neurobiolaging.2007.08.022 PMC3164546

[CIT0008] Lant S. B., Robinson A. C., Thompson J. C., Rollinson S., Pickering-Brown S., Snowden J. S., … Mann D. M. A. (2014). Patterns of microglial cell activation in frontotemporal lobar degeneration. *Neuropathology and Applied Neurobiology*, 40, 686–696. 24117616 10.1111/nan.12092

[CIT0009] Lashley T., Holton J. L., Gray E., Kirkham K., O’Sullivan S. S., Hilbig A., … Revesz T. (2008). Cortical alpha-synuclein load is associated with amyloid-beta plaque burden in a subset of Parkinson’s disease patients. *Acta Neuropathologica*, 115, 417–425. 18185940 10.1007/s00401-007-0336-0

[CIT0010] Le Ber I., Camuzat A., Hannequin D., Pasquier F., Guedj E., Rovelet-Lecrux A., … Camu W. (2008). Phenotype variability in progranulin mutation carriers: A clinical, neuropsychological, imaging and genetic study. *Brain*, 131, 732–746. 18245784 10.1093/brain/awn012

[CIT0011] Lui H., Zhang J., Makinson S. R., Cahill M. K., Kelley K. W., Huang H. Y., … Huang E. J. (2016). Progranulin deficiency promotes circuit-specific synaptic pruning by microglia via complement activation. *Cell*, 165, 921–935. 27114033 10.1016/j.cell.2016.04.001 PMC4860138

[CIT0012] Mackenzie I. R. A., Neumann M., Baborie A., Sampathu D. M., Du Plessis D., Jaros E., … Lee V. M. Y. (2011). A harmonized classification system for FTLD-TDP pathology. *Acta Neuropathologica*, 122, 111–113. 21644037 10.1007/s00401-011-0845-8 PMC3285143

[CIT0013] Martens L. H., Zhang J., Barmada S. J., Zhou P., Kamiya S., Sun B., … Farese R. V. (2012). Progranulin deficiency promotes neuroinflammation and neuron loss following toxin-induced injury. *Journal of Clinical Investigation*, 122, 3955–3959. 23041626 10.1172/JCI63113 PMC3484443

[CIT0014] McAleese K. E., Firbank M., Dey M., Colloby S. J., Walker L., Johnson M., … Attems J. (2015). Cortical tau load is associated with white matter hyperintensities. *Acta Neuropathologica Communications*, 3, 60. 26419828 10.1186/s40478-015-0240-0 PMC4589169

[CIT0015] McAleese K. E., Walker L., Graham S., Moya E. L. J., Johnson M., Erskine D., … Attems J. (2017). Parietal white matter lesions in Alzheimer’s disease are associated with cortical neurodegenerative pathology, but not with small vessel disease. *Acta Neuropathologica*, 134, 459–473. 28638989 10.1007/s00401-017-1738-2 PMC5563333

[CIT0016] McMillan C. T., Irwin D. J., Avants B. B., Powers J., Cook P. A., Toledo J. B., … Grossman M. (2013). White matter imaging helps dissociate tau from TDP-43 in frontotemporal lobar degeneration. *Journal of Neurology, Neurosurgery and Psychiatry*, 84, 949–955. 10.1136/jnnp-2012-304418 PMC3737288 23475817

[CIT0017] Minett T., Classey J., Matthews F. E., Fahrenhold M., Taga M., Brayne C., & Cfas M. R. C. (2016). Microglial immunophenotype in dementia with Alzheimer’s pathology. *Journal of Neuroinflammation*, 13, 135. 27256292 10.1186/s12974-016-0601-z PMC4890505

[CIT0018] Murray M. E., Vemuri P., Preboske G. M., Murphy M. C., Schweitzer K. J., Parisi J. E., … Dickson D. W. (2012). A quantitative postmortem MRI design sensitive to white matter hyperintensity differences and their relationship with underlying pathology. *Journal of Neuropathology & Experimental Neurology*, 71, 1113–1122. 23147507 10.1097/NEN.0b013e318277387e PMC3511604

[CIT0019] Paternicò D., Premi E., Gazzina S., Cosseddu M., Alberici A., Archetti S., … Borroni B. (2016). White matter hyperintensities characterize monogenic frontotemporal dementia with granulin mutations. *Neurobiology of Aging*, 38, 176–180. 26827655 10.1016/j.neurobiolaging.2015.11.011

[CIT0020] Pietroboni A. M., Fumagalli G. G., Ghezzi L., Fenoglio C., Cortini F., Serpente M., … Scarpini E. (2011). Phenotypic heterogeneity of the GRN Asp22fs mutation in a large italian kindred. *Journal of Alzheimer’s Disease*, 24, 253–259. 10.3233/JAD-2011-101704 21258152

[CIT0021] Ryan N. S., Biessels G.-J., Kim L., Nicholas J. M., Barber P. A., Walsh P., … Fox N. C. (2015). Genetic determinants of white matter hyperintensities and amyloid angiopathy in familial Alzheimer’s disease. *Neurobiology of Aging*, 36, 3140–3151. 26410308 10.1016/j.neurobiolaging.2015.08.026

[CIT0022] Sarbu N., Shih R. Y., Jones R. V., Horkayne-Szakaly I., Oleaga L., & Smirniotopoulos J. G. (2016). White matter diseases with radio- logic-pathologic correlation. *RadioGraphics*, 36, 1426–1447. 27618323 10.1148/rg.2016160031

[CIT0023] Shim Y. S., Yang D.-W., Roe C. M., Coats M. A., Benzinger T. L., Xiong C., … Morris J. C. (2015). Pathological correlates of white matter hyperintensities on magnetic resonance imaging. *Dementia and Geriatric Cognitive Disorders*, 39, 92–104. 25401390 10.1159/000366411 PMC4312498

[CIT0024] Skrobot O. A., Attems J., Esiri M., Hortobagyi T., Ironside J. W., Kalaria R. N., … Love S. (2016). Vascular cognitive impairment neuropathology guidelines (VCING): The contribution of cerebrovascular pathology to cognitive impairment. *Brain*, 139, 2957–2969. 27591113 10.1093/brain/aww214

[CIT0025] Snyder H. M., Corriveau R. A., Craft S., Faber J. E., Greenberg S. M., Knopman D., … Carrillo M. C. (2015). Vascular contributions to cognitive impairment and dementia including Alzheimer’s disease. *Alzheimer’s & Dementia : the Journal of the Alzheimer’s Association*, 11, 710–717. 10.1016/j.jalz.2014.10.008 PMC4731036 25510382

[CIT0026] Streit W. J., Braak H., Xue Q.-S., & Bechmann I. (2009). Dystrophic (senescent) rather than activated microglial cells are associated with tau pathology and likely precede neurodegeneration in Alzheimer’s disease. *Acta Neuropathologica*, 118, 475–485. 19513731 10.1007/s00401-009-0556-6 PMC2737117

[CIT0027] Streit W. J., Xue Q.-S., Tischer J., & Bechmann I. (2014). Microglial pathology. *Acta Neuropathologica Communications*, 2, 142. 25257319 10.1186/s40478-014-0142-6 PMC4180960

[CIT0028] Sudre C. H., Bocchetta M., Cash D., Thomas D. L., Woollacott I., Dick K. M., … Rohrer J. D. (2017). White matter hyperintensities are seen only in GRN mutation carriers in the GENFI cohort. *NeuroImage: Clinical*, 15, 171–180. 28529873 10.1016/j.nicl.2017.04.015 PMC5429247

[CIT0029] Sudre C. H., Cardoso M. J., Bouvy W. H., Biessels G. J., Barnes J., & Ourselin S. (2015). Bayesian model selection for pathological neuroimaging data applied to white matter lesion segmentation. *IEEE Transactions on Medical Imaging*, 34, 2079–2102. 25850086 10.1109/TMI.2015.2419072

[CIT0030] Sudre C. H., Cardoso M. J., & Ourselin S. (2017). Longitudinal segmentation of age-related white matter hyperintensities. *Medical Image Analysis*, 38, 50–64. 28282640 10.1016/j.media.2017.02.007

[CIT0031] Taipa R., Brochado P., Robinson A., Reis I., Costa P., Mann D. M., … Sousa N. (2017). Patterns of microglial cell activation in alzheimer disease and frontotemporal lobar degeneration. *Neurodegenerative Diseases*, 17, 145–154. 28445885 10.1159/000457127

[CIT0032] Tischer J., Krueger M., Mueller W., Staszewski O., Prinz M., Streit W. J., & Bechmann I. (2016). Inhomogeneous distribution of Iba-1 characterizes microglial pathology in Alzheimer’s disease. *Glia*, 64, 1562–1572. 27404378 10.1002/glia.23024

[CIT0033] Tofts P. S., Jackson J. S., Tozer D. J., Cercignani M., Keir G., MacManus D. G., … Fox N. C. (2008). Imaging cadavers: Cold FLAIR and noninvasive brain thermometry using CSF diffusion. *Magnetic Resonance in Medicine*, 59, 190–195. 18058937 10.1002/mrm.21456 PMC2478723

[CIT0034] Wardlaw J. M., Valdés Hernández M. C., & Muñoz-Maniega S. (2015). What are white matter hyperintensities made of? Relevance to vascular cognitive impairment. *Journal of the American Heart Association*, 4, 1140. 10.1161/JAHA.114.001140 PMC4599520 26104658

[CIT0035] Yin F., Banerjee R., Thomas B., Zhou P., Qian L., Jia T., … Ding A. (2009). Exaggerated inflammation, impaired host defense, and neuropathology in progranulin-deficient mice. *Journal of Experimental Medicine*, 207, 117–128. 20026663 10.1084/jem.20091568 PMC2812536

[CIT0036] Yin F., Dumont M., Banerjee R., Ma Y., Li H., Lin M. T., … Ding A. (2010). Behavioral deficits and progressive neuropathology in progranulin-deficient mice: A mouse model of frontotemporal dementia. *FASEB Journal : Official Publication of the Federation of American Societies for Experimental Biology*, 24, 4639–4647. 20667979 10.1096/fj.10-161471 PMC2992364

