# Supplementary material for: Pathological correlates of white matter hyperintensities in a case of progranulin mutation associated frontotemporal dementia
Source: Neurocase. 2018 Aug 16;24(3):166–74. doi: 10.1080/13554794.2018.1506039 (PMC6168954; doi:10.1080/13554794.2018.1506039)
Supplement: Supplemental Material [file NNCS_A_1506039_SM7048.docx]

**Supplementary Material**

**Imaging analysis methods**

To allow regional quantification of WMH, segmentation of WMH was first performed using the cadaveric MRI FLAIR sequences. WMH were automatically segmented using an algorithm based on outlier modelling in a multivariate Gaussian mixture model as described previously (Sudre et al., 2015). Localization of WMH was then performed by apportioning them into zones, based on whether they were nearest to a cortical lobe, basal ganglia, or the thalami, as described previously (Sudre, Cardoso, & Ourselin, 2017). The localization was further discretized into four equidistant brain layers based on relative distance between ventricles and cortical sheet (first layer most periventricular, fourth layer most juxtacortical), and to the closest cortical lobe. Infratentorial regions were excluded from analysis and these did not display WMH on MRI. Localization and discretization analysis produced two measures of WMH burden for the cadaveric MRI: lesion distribution (volume of lesion in a region/total volume of lesion in the brain; i.e. the proportion of the total brain WMH lesion load present in that particular region) and regional lesion frequency (volume of lesion in a region/volume of this region; i.e. the proportion of the volume of a region taken up by lesion). This allowed quantification of WMH burden in each lobe and within different cortical and subcortical ‘layers’ in each lobe, to guide selection of tissue slices and location of tissue blocks corresponding to different severities of WMH on MRI, for detailed histological analysis.

**Immunohistochemistry methods**

Immunohistochemistry was performed using antibodies to detect the presence of TDP-43 (Proteintech; dilution 1:800), Aβ (Dako; dilution 1:100), tau (AT8 antibody, Thermo Scientific; dilution 1:600), alpha synuclein (BD Bioscience; dilution 1:1000), phosphorylated neurofilaments for axons (SMI31 antibody, Sternberger Monoclonals; dilution 1:5000) and activated astrocytes (glial fibrillary acidic protein [GFAP] antibody, Dako; dilution 1:1000), as well as three different microglial markers: CD68 (Dako; dilution 1:100), CR3/43 (Dako, dilution 1:150) and Iba1 (Wako Chemicals; dilution 1:1000). Sections were dewaxed in xylene and rehydrated in graded alcohol solutions, endogenous peroxidase activity blocked using 0.3% H_2_O_2_ (BDH) in methanol for ten minutes, and non-specific binding blocked with 10% non-fat milk in TBS for 30 minutes. Immunohistochemistry for all antibodies required pre-treatment in citrate buffer (pH 6.0) using a pressure cooker for 10 minutes and additional pre-treatment with formic acid for Aβ. Tissue sections were incubated in primary antibodies for one hour at room temperature, then washed in TBS and incubated in biotinylated anti-rabbit IgG (Dako; dilution 1:200) or biotinylated anti-mouse IgG (Dako; dilution 1:200) secondary antibodies for 30 minutes. Following further TBS washes, sections were incubated in Avidin–Biotin Complex (ABC Kit, Vector Laboratories Inc.) for 30 minutes, and color developed with 3,3’-diaminobenzidine (DAB, Sigma) as chromogen and H_2_O_2_ as substrate. Sections were then dehydrated using graded alcohol solutions, cleared in xylene and mounted using DePeX (BDH).

**Supplementary Table 1.** Parameters of vascular pathology analysed based on recommendations of the Vascular Cognitive Impairment Neuropathology Guidelines for staging of cerebrovascular pathology in cognitive impairment (Skrobot et al., 2016).

| **Vascular changes scoring** | **Vessel wall pathology scoring** |
| --- | --- |
| **Myelin loss**  0 = dense and homogeneous myelin staining  1 = mild diffuse or focal myelin pallor  2 = severe focal/diffuse myelin pallor with vacuolation or tigroid appearance of the white matter  3 = total focal/diffuse destruction of the myelin, or white matter infarcts | **Arteriolosclerosis (vessels < 150 µm diameter)**  0 = normal  1 = mild hyaline thickening of the vessel media, mild fibrosis  2 = partial loss of smooth muscle cells in the media, moderate hyaline fibrosis  3 = complete loss of smooth muscle cells in the media, severe hyaline fibrosis, lumen stenosis |
| **Perivascular space dilatation**  0 = absent  1 = perivascular space < artery diameter in all sections  2 = perivascular space ≥ artery diameter in the minority of sections  3 = perivascular space ≥ artery diameter in the majority of sections | **Fibrinoid necrosis or microaneurysms**  0 = absent  1 = present |
| **Perivascular hemosiderin leakage**  0 = absent  1 = <3 hemosiderin granule deposits in the perivascular space  2 = 3-5 hemosiderin granule deposits in the perivascular space  3 = >5 hemosiderin granule deposits in the perivascular space | **Cerebral amyloid angiopathy (leptomeningeal, cortical and capillary)**  0 = absent  1= trace or occasional vessels affected  2 = one or a few vessels circumferentially affected  3 = widespread involvement of circumferentially affected vessels  4 = as 3, with secondary changes |
| **Microinfarcts**  0 = absent  1 = present | **Circle of Willis atherosclerosis**  0 – no atheroma  1 – atheroma present but no artery >50% occluded  2 – atheroma and one vessel ≥50% occluded  3- atheroma and >1 vessel ≥50% occluded |
| **Large infarcts**  0 = absent  1 = present |  |
| **Lacunar infarcts**  0 = absent  1 = solitary  2 = 2-4  3 = 5 or more |  |
| **Microhemorrhage**  0 = absent  1 = present |  |
| **Larger hemorrhage**  0 = absent  1 = present |  |
